# A novel small molecule LLL12B inhibits STAT3 signaling and sensitizes ovarian cancer cell to paclitaxel and cisplatin

**DOI:** 10.1371/journal.pone.0240145

**Published:** 2021-04-28

**Authors:** Ruijie Zhang, Xiaozhi Yang, Dana M. Roque, Chenglong Li, Jiayuh Lin

**Affiliations:** 1 Department of Thoracic Surgery, Tongji Hospital, Tongji Medical College, Huazhong University of Science and Technology, Wuhan, P.R. China; 2 Department of Biochemistry and Molecular Biology, University of Maryland School of Medicine, Baltimore, Maryland, United States of America; 3 Department of Medicinal Chemistry, College of Pharmacy, The University of Florida, Gainesville, Florida, United States of America; 4 Division of Gynecologic Oncology, Department of Obstetrics and Gynecology, University of Maryland School of Medicine, Baltimore, Maryland, United States of America; Duke University School of Medicine, UNITED STATES

## Abstract

Ovarian cancer is the fifth most common cause of cancer deaths among American women. Platinum and taxane combination chemotherapy represents the first-line approach for ovarian cancer, but treatment success is often limited by chemoresistance. Therefore, it is necessary to find new drugs to sensitize ovarian cancer cells to chemotherapy. Persistent activation of Signal Transducer and Activator of Transcription 3 (STAT3) signaling plays an important role in oncogenesis. Using a novel approach called advanced multiple ligand simultaneous docking (AMLSD), we developed a novel nonpeptide small molecule, LLL12B, which targets the STAT3 pathway. In this study, LLL12B inhibited STAT3 phosphorylation (tyrosine 705) and the expression of its downstream targets, which are associated with cancer cell proliferation and survival. We showed that LLL12B also inhibits cell viability, migration, and proliferation in human ovarian cancer cells. LLL12B combined with either paclitaxel or with cisplatin demonstrated synergistic inhibitory effects relative to monotherapy in inhibiting cell viability and LLL12B-paclitaxel or LLL12B-cisplatin combination exhibited greater inhibitory effects than cisplatin-paclitaxel combination in ovarian cancer cells. Furthermore, LLL12B-paclitaxel or LLL12B-cisplatin combination showed more significant in inhibiting cell migration and growth than monotherapy in ovarian cancer cells. In summary, our results support the novel small molecule LLL12B as a potent STAT3 inhibitor in human ovarian cancer cells and suggest that LLL12B in combination with the current front-line chemotherapeutic drugs cisplatin and paclitaxel may represent a promising approach for ovarian cancer therapy.

## Introduction

Ovarian cancer is the most lethal gynecologic malignancy [1, 2]. In 2018, there were approximately 22,240 new cases and 14,070 deaths from ovarian cancer in the United States [3]. Because of a lack of early symptoms, nearly 80% of patients will be diagnosed at an advanced stage [1]. Adjuvant chemotherapy is usually needed following surgical cytoreduction. Platinum in combination with taxane chemotherapy is considered a first-line approach [4, 5]. Unfortunately, five-year survival rates have not improved much during the past 20 years [6, 7], due to both intrinsic and acquired chemoresistance. Many research efforts focus upon reversal of chemotherapy resistance for recurrent disease, but less attention is given to enhancing sensitivity to chemotherapy during the primary treatment of ovarian cancer [8–11].

In recent years, many researchers have invested in biotherapies with more precise targets, including immunotherapy, gene therapy, and molecular targeted therapy, which may be more effective and less toxic [12–16]. As the most common member of the signal transducers and activators of transcription (STAT) proteins, STAT3 is critical to many signaling pathways [17, 18]. In normal cells, the activation of STAT3 is transitory and restricted; it can promote embryo development and growth, induce inflammation, and cause autophagy, among other processes [19]. STAT3 also plays an important role in the development of tumors and is now considered an oncogene. The constitutive and abnormal activation of STAT3 can upregulate or downregulate many target tumor-related genes, such as *BCL-2*, *c-myc*, *cyclinD1*, *survivin*, *cleaved caspase-3*, *HIF-1* and *VEGF*, which then enable various processes key to malignant progression, such as cell proliferation, tumor initiation, migration, invasion, angiogenesis, metastasis, cell cycle dysregulation, induction of the epithelial mesenchymal transition (EMT), and inhibition of apoptosis, as well as promote multidrug resistance to chemotherapy [19].

STAT3 activation occurs when the tyrosine 705 (Tyr705) residue is phosphorylated. Using a novel approach called advanced multiple ligand simultaneous docking (AMLSD), we developed several new small molecular inhibitors targeting STAT3, including the novel STAT3 inhibitor LLL12B. Computer models with docking simulation showed that LLL12B binds directly to the phosphoryl tyrosine 705 (pTyr705) binding site of the STAT3 monomer. In the present study, we characterized the biologic effects of LLL12B alone and in combination with chemotherapy on ovarian cancer cells.

## Material and methods

### Materials

The small molecule LLL12B, a novel STAT3 inhibitor, was synthesized at University of Florida College of Pharmacy (Chenglong Li) [20]. LLL12B powder was dissolved in sterile dimethyl sulfoxide (DMSO) to make a 20 mM stock solution and stored at -20 °C. Cisplatin and 3-(4, 5-dimethylthiazol-2-yl)-2, 5-diphenyltetrazolium bromide (MTT) were purchased from Sigma (Burlington, MA). The stock concentration of cisplatin was 5mM in ddH2O. Paclitaxel was obtained from LC Laboratories (Woburn, MA). The stock concentration of paclitaxel was 20mM in DMSO. Primary and secondary antibodies were bought from Cell Signaling Technology (Danvers, MA).

### Synthesis of LLL12B

Firstly, 1-naphthalenesulfonyl chloride (1, 50 g; what does it mean? 50 g?) was stirred with 28% ammonium hydroxide (300 mL) in acetone (1 L) at rt for about 3 h, concentrated, cooled to rt (room temperature, filtered, washed and dried by air, 42 g of white powder 1-naphthalenesulfonamide (2) was obtained. Secondly, the compound 2 (48 g) was dissolved completely suspended in acetic acid (480 mL) andthen cooled to 40 ~ 45°C, and CrO3 (104 g) solution in H2O (100 mL) and acetic acid (100 mL) was added in 1 ~ 1.5 h, which was then stirred, filtered, washed and dried by air to obtainthe crude product (24 g). 48g crude product was dissolved in minimum acetoneat rt and hexane was added slowly till precipitate was just observed, then place it in refrigerator (about -20°C) overnight. Filtration afforded 26.9 g of compound 3. Thirdly, the compound 3 (15.45 g) was dissolved in CH2Cl2 (1.2 L) and methanol (162 mL) at rt, then cooled to -20 ~ -15°C and Et3N (1.54 mL) was added. After stirring for about 15 min, 3-hydroxy-1-pyrone (8.74 g) in 300 mL of CH2Cl2 was added and stirred for about 30 min, then 2 ~ 3 h at rt. The formed yellow precipitate was collected by filter to get the first crop of LLL12 (3.24 g). Filtrate was concentrated at 33°C by rotary evaporation under hose vacuum to 150 ml. Add 250 ml CH2Cl2 to precipitate, filter and wash with small quantity CH2Cl2 to get 2nd crop of LLL12(5.27g). Further purification by silica gel flash column chromatography eluted with mixed solvents of acetone and hexanes (1:1, V/V) afforded 3.95 g of LLL12. Finally, LLL12 (2.01 mg) was suspended in pyridine (15 g) at rt, then dimethycarbamyl chloride (1.02 g) was added and stirred at rt overnight. Then the reaction mixtures were filtered and washed with CH2Cl2 and then large quantity of acetone (about 1 L) to afford the compound of LLL12B.

### Cell lines

All four human ovarian cancer cell lines (A2780, SKOV3, CAOV3, and OVCAR5), were purchased from ATCC (American Type Culture Collection, Manassas, VA). OVAR5 was cultured in RPMI1640 medium with 10% fetal bovine serum (FBS) and 1% penicillin/ streptomycin(PS); A2780, CAOV3, and SKOV3 were cultured in Dulbecco’s modified Eagle medium (DMEM) with 10% FBS and 1% PS. All of the cell lines were maintained in a humidified 37°C incubator with 5% CO_2_/95% air. Media were replaced twice a week.

### Western blot analysis

The four cell lines were seeded in 10cm-plates in 70% cell density, cultured overnight, then treated with DMSO, cisplatin, paclitaxel, different concentrations of LLL12B and their combination or treated with LLL12B by 0h,2h and 4h. Cells were cultured overnight before they were harvested for Western blot analysis. Ovarian cancer cells were lysed in cold lysis buffer and the proteins were separated by 10% SDS-PAGE. Proteins were transferred to PVDF membrane under 350mA for 110 minutes and then blocked by 5% milk for 1 hour and incubated overnight at 4°C with primary antibodies: all the primary antibodies are rabbit mAbs and bought from Cell Signaling Technology, Inc; Anti-phosphorylated (p)-STAT3 (Tyr705) (cat. no. NC-CST-9145S), STAT3 (cat. no. 4904), GAPDH (cat. no. 2118S), c-Myc(cat. no. 9402s), cyclinD1(cat. no. 55506s), survivin(cat. no. 2808s), and cleaved caspase 3(cat. no. 9661s). All primary antibodies were diluted 1:1,000 in 5% milk. After washing with tris-buffered saline-tween (TBST) for 3 times (15 min), the membranes were blotted with the horseradish peroxidase-conjugated anti-rabbit IgG secondary antibody diluted 1:10,000 in 5% milk (Cell Signaling Technology, Inc.; cat. no. 7074) at room temperature for 1.5 h. The p-STAT3 protein were detected by West Femto Maximum Sensitivity Substrate (Thermo Fisher Scientific, Inc; cat. no. 34096); the STAT3 and GAPDH proteins were detected by enhanced chemiluminescence substrate (PerkinElmer, Inc; cat. no. NEL104001EA). Blots were imaged with the Storm scanner (Amersham Pharmacia Biotech Inc, Piscataway, NJ, USA) and Amersham Imager 680(GE healthcare Inc, Mariborough, MA, USA).

### MTT cell viability assay

We seeded A2780, SKOV3, CAOV3 and OVCAR5 in 96-well microtiter plates at a density of 3,000 cells with 100μl medium per well. After overnight incubation, the cells were treated in each well at 37°C with vehicle control (DMSO) or different concentrations of drugs: LLL12B, cisplatin alone, paclitaxel alone, or their combination. Seventy-two hours later, we added 20μl of MTT to each well. After incubation for 4 h at 37°C, each well was supplemented with 150μl of dimethylformamide solubilization solution followed by an incubation overnight, protected from light at room temperature. Cell viability was assessed using the absorbance at 595 nm of each well. The DMSO cells were set at 100% and the cell viability of drug-treated cells was determined relative to DMSO cells. Then the combination index (CI) was determined by CompuSyn software (www.combosyn.com). The CI values indicate an additive effect when equal to 1, an antagonistic effect when >1, and a synergistic effect when <1 based on the theorem of Chou and Talalay [21].

### Wound-healing/cell migration assay

A2780 and SKOV3 cells were seeded in 6-well plates and incubated at 37°C overnight. When cells reached 100% confluence, the monolayer was scratched by a 100-μl pipette tip. We washed each well with PBS twice and added new medium with different drugs: DMSO, LLL12B, paclitaxel, cisplatin or their combination. Photos of each well were captured by microscope at time zero. Cells were incubated at 37°C until the wound of the control well was healed (SKOV3, 18h; A2780, 56h). The photos of each well were captured by microscope again after washing in PBS twice. Inhibition of migration was measured by ImageJ software (http://rsb.info.nih.gov/ij/) and calculated by the formula: percent of wound healed = 100 − [(final area / initial area) × 100%] [22].

### Cell growth assay

All four cell lines were seeded in 12-well plates at the same cell density, which was dependent on the growth ability of each cell line (SKOV3:1*10^4^ cells per well, CAOV3: 2.5*10^4^ cells per well, A2780:0.5*10^4^ cells per well, and OVCAR5: 1*10^4^ cells per well). The cells were cultured overnight at 37°C, then were treated with DMSO or different concentrations of drugs: LLL12B, paclitaxel alone, cisplatin alone, or the combination. We counted the cell number of each well days 2, 4, and 6 after treatment to generate growth curves. Significant differences were defined as **p<0.01 and ****p<0.0001.

### Statistical analysis

Significance of correlations was determined by GraphPad Prism 7 software (GraphPad Software Inc., San Diego, CA, USA). The data were expressed as mean ± standard deviations (SD). One-way ANOVA and two-way ANOVA with Tukey’s Test were used to analyze the statistical difference between two groups. Significance was set at *p* < 0.05. The *, ** and *** indicate *p* < 0.05, *p* < 0.01 and *p* < 0.001, respectively.

## Results

### LLL12B inhibited STAT3 phosphorylation and the expression of downstream targeted genes

**Fig 1:**
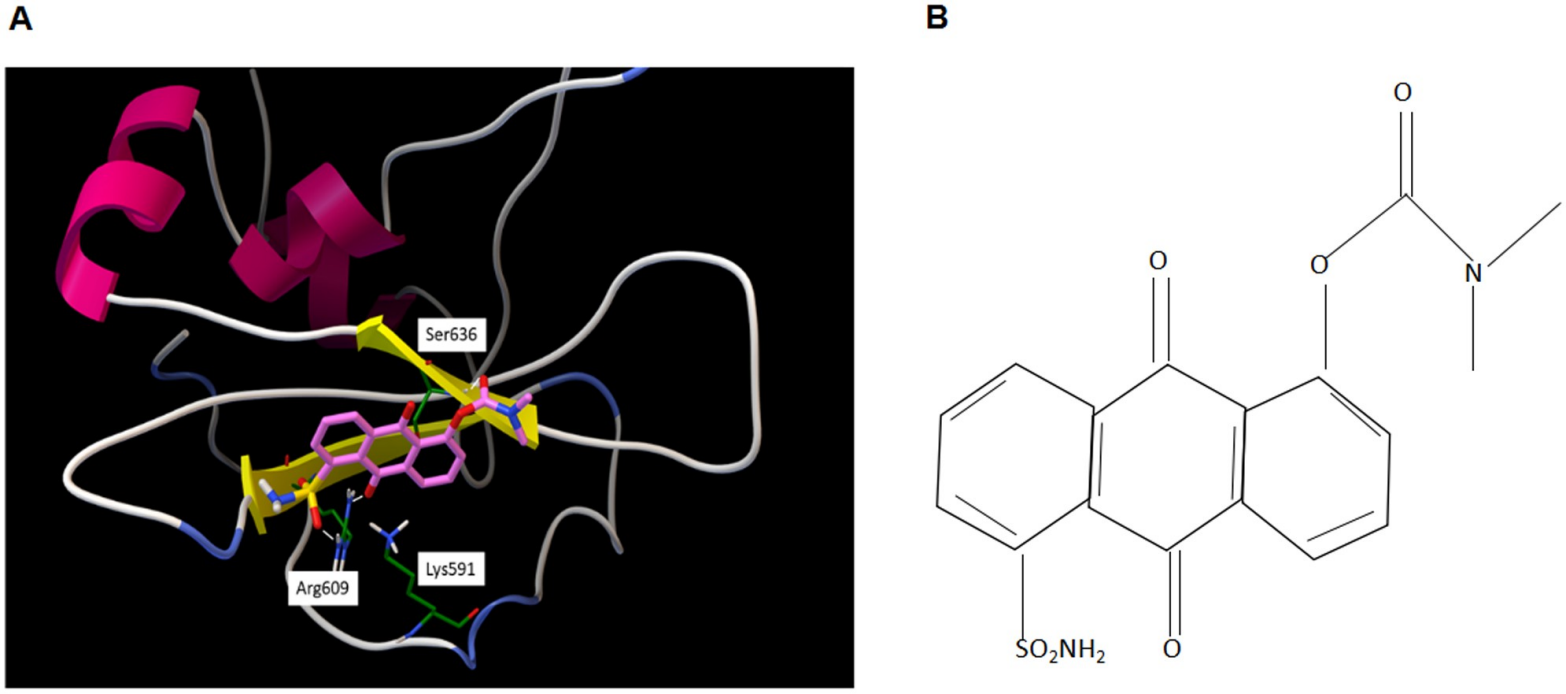
The computational model and chemical structure of LLL12B. A. The computational model of LLL12B. B. The chemical structure of LLL12B.

**Fig 2:**
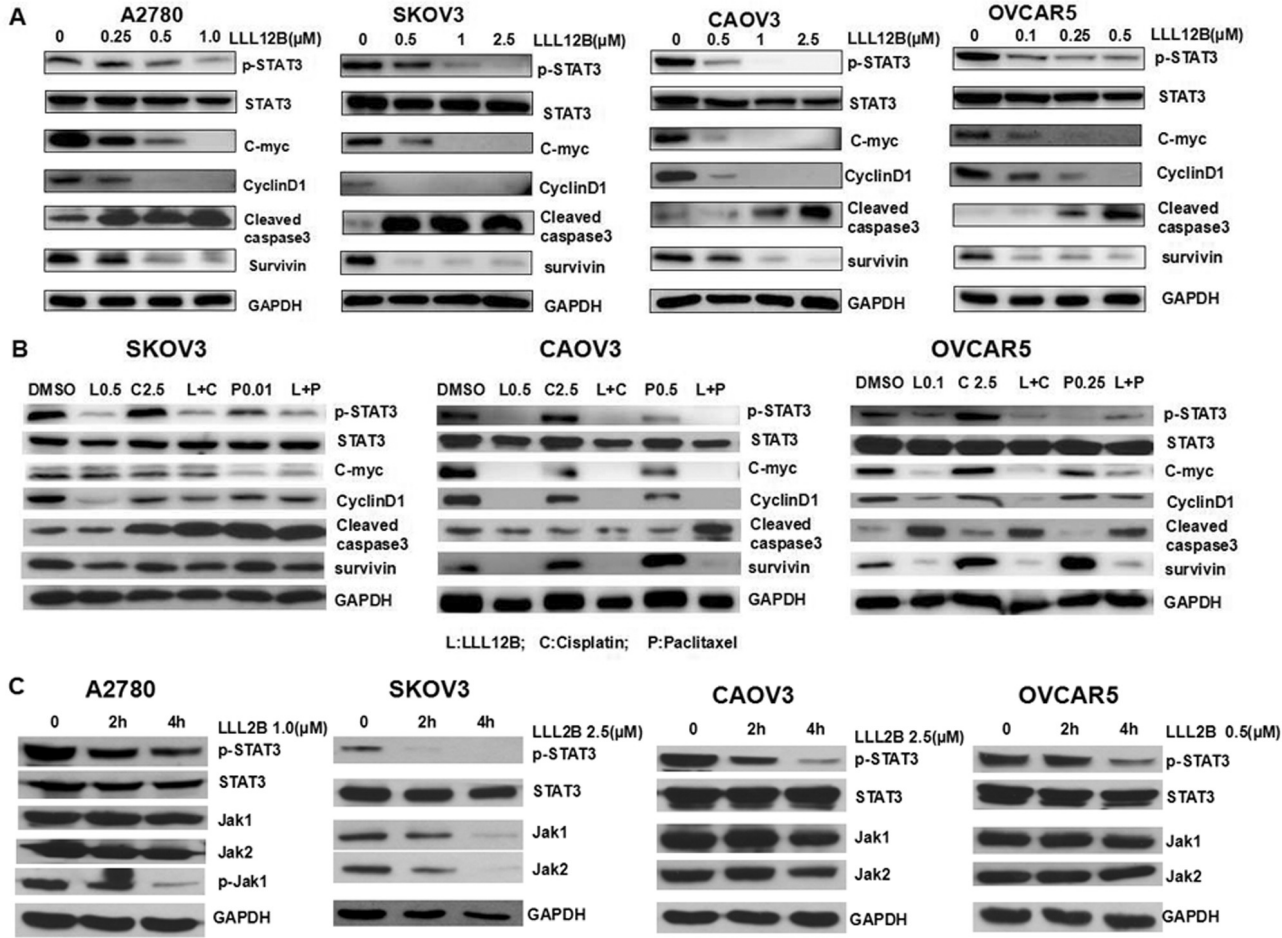
LLL12B inhibits p-STAT3 and its downstream targets in human ovarian cancer cells. A. The effects of LLL12B on STAT3 phosphorylation (Tyr705) and its downstream targets in ovarian cancer cell lines. A2780, CAOV3, SKOV3, OVCAR5 cells were treated with DMSO or different concentrations of LLL12B. The levels of p-STAT3 and the downstream target gene proteins were determined by Western blots. B. Compared with cisplatin or paclitaxel alone, the combination of LLL12 with cisplatin or/and paclitaxel appeared to have a greater inhibitory effect on p-STAT3 and some downstream target gene proteins in CAOV3, SKOV3 and OVCAR5 cells. C. Treated with LLL12B by 0h,2h and 4h, p-STAT3 was inhibited in four cell lines, p-Jak1 was only inhibited in A2780; p-Jak2 was not detected. Total Jak1, Jak2 in SKOV3 was reduced. The total STAT3 and total Jak1, Jak2 in other three cell lines were not reduced.

To target STAT3, we used a novel approach called advanced multiple ligand simultaneous docking (AMLSD), and developed a novel nonpeptide small molecule, LLL12B. The computational model figure showed LLL12B binding to STAT3 SH2 domain (Fig 1A): Arg609 forms two hydrogen bonds with LLL12B; Ser636 forms one hydrogen bond; and Lys591 has cation-pi interaction with LLL12B. The chemical structure of LLL12B was shown as Fig 1B. In order to examine the ability of LLL12B to inhibit p-STAT3 (Tyr705) *in vitro*, Western blotting analysis was performed by us. All four ovarian cancer cell lines were seeded in 10 cm plates and were treated with DMSO or different concentrations of LLL12B (0.25uM-2.5uM). Protein expression levels were analyzed. Compared with those treated by DMSO, p-STAT3 was inhibited by LLL12B (Fig 2A). In addition, the STAT3 downstream targets c-myc, cyclinD1, and survivin were down-regulated and cleaved caspase-3 was induced (Fig 2A). LLL12B inhibited STAT3 phosphorylation and down-regulated downstream target genes which are associated with cancer cell proliferation and growth [23–26]. Compared with cisplatin or paclitaxel alone, the combination of LLL12 with cisplatin or/and paclitaxel led to a greater inhibitory effect on p-STAT3 and some downstream targets (Fig 2B):Cisplatin and paclitaxel cisplatin or paclitaxel alone are in general not able to significantly inhibit C-Myc and CyclinD1, which were reduced by combination with LLL12B; cleaved caspase3 was induced by cisplatin and paclitaxel and enhanced by combination with LLL12B; cisplatin and paclitaxel upregulated or didn’t affect the expression of survivin, which was reduced by combination with LL12B. These results indicate that LLL12B is a biologically relevant potent STAT3 inhibitor of ovarian cancer cells.

Then, we performed the WB assay at 2 hours and 4 hours after LLL12B was added. The results showed that (Fig 2C) p-STAT3 was inhibited in four cell lines, the p-Jak1 was only inhibited in A2780, however the p-Jak1 in other three cell lines and p-Jak2 was not detected. Total Jak1, Jak2 in SKOV3 was reduced. The total STAT3 and total Jak1, Jak2 in other three cell lines were not reduced. The results showed that p-STAT3 was definitely inhibited in all the four cell lines which may be the main function of LLL12B. It may be possible that LLL12B is a dual inhibitor of STAT3 and Jak1 in certain cell lines just like a few other STAT3 inhibitors [27].

### LLL12B inhibited cell viability of human ovarian cancer cells and synergistically enhanced the effect of cisplatin and paclitaxel

**Fig 3:**
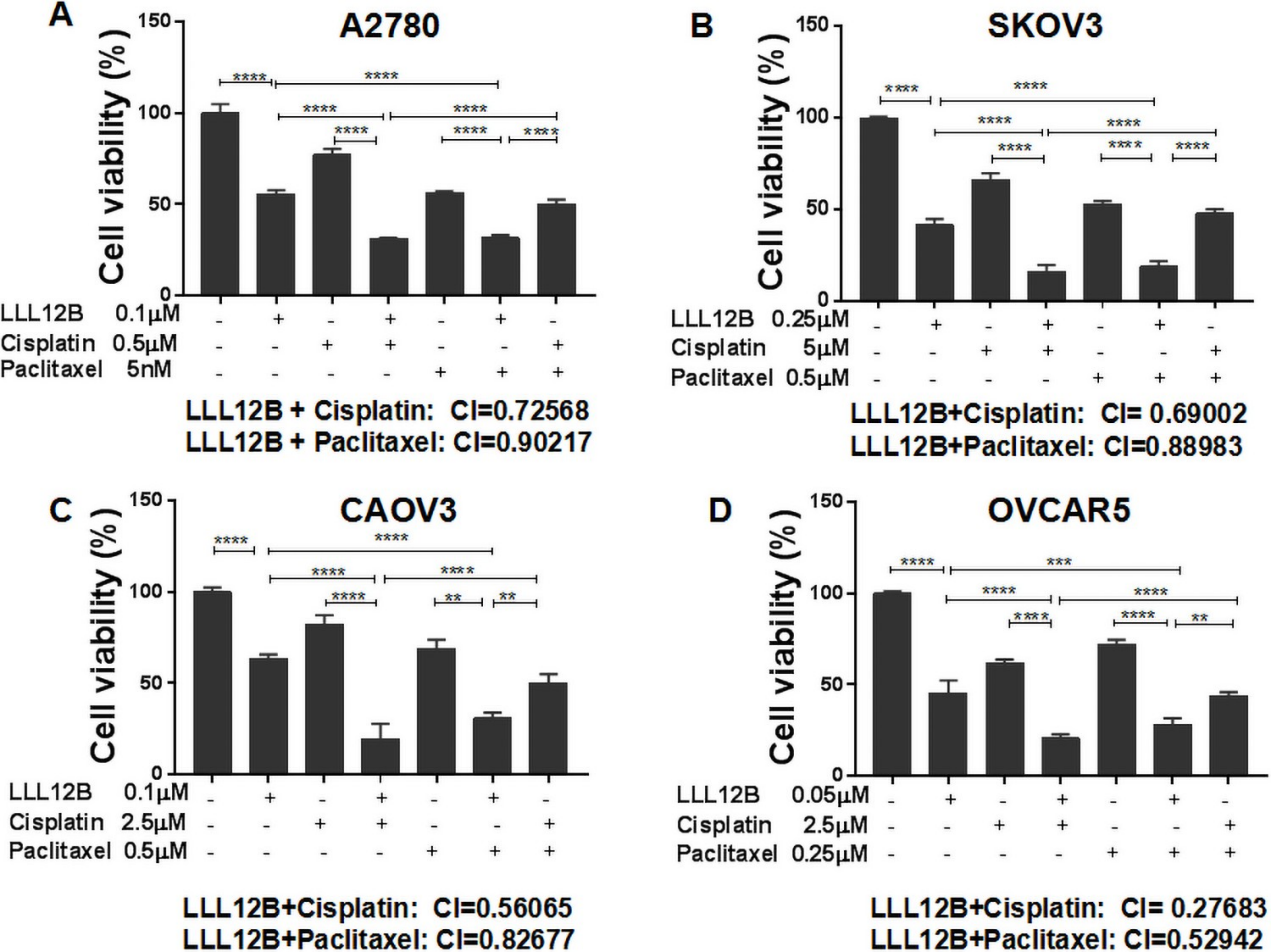
The effects of LLL12B, cisplatin, paclitaxel, and drug combination on cell viability. MTT assays were performed to evaluate cell viability. A-D LLL12B inhibited cell viability of ovarian cancer cells, which were synergistically inhibited when LLL12B was combined with cisplatin or paclitaxel. The differences were found to be significantly different at *p<0.05, **p<0.01, ***p<0.001 and ****p<0.0001.

To evaluate inhibition of cell viability, MTT assays were performed using A2780, CAOV3, SKOV3 and OVCAR5 cells. Cells were seeded in 96-well plates and treated with LLL12B or DMSO control followed by culture at 37°C for additional 72 hours. Cell viability was significantly inhibited by LLL12B (Fig 3). To investigate whether chemotherapy can be enhanced by LLL12B, the cells were treated by LLL12B combined with cisplatin or paclitaxel. The combination index (CI) showed that the suppression achieved with combination treatment was more significant than that of any monotherapy. The CIs of LLL12B combined with cisplatin or paclitaxel in each cell line were all less than 1(Fig 3 and Table 1), which indicated synergism. Furthermore, the combination of LLL12B with cisplatin or paclitaxel exhibited more significant inhibitory effects on cell viability than the combination of cisplatin and paclitaxel (Fig 3). These results indicate that LLL12B inhibits cell viability and also synergistically enhances the effect of chemotherapy in ovarian cancer cell lines.

**Table 1:** The calculation of the combination index (CI).

	LLL12B		Cisplatin		Paclitaxel		LLL12B+Cisplatin				LLL12B+Paclitaxel			
	Dose (μM)	Effect	Dose (μM)	Effect	Dose (μM)	Effect	Dose L (μM)	Dose C (μM)	Effect	CI	Dose L (μM)	Dose P (μM)	Effect	CI
**A2780**	0.05	0.906	0.5	0.77	0.005	0.56	0.1	0.5	0.31	0.72568	0.1	0.0025	0.384	0.94247
	0.1	0.577	1.0	0.7	0.0025	0.66	0.1	1.0	0.245	0.65333	0.1	0.005	0.3166	0.90217
**SKOV3**	0.25	0.415	2.5	0.751	0.25	0.676	0.25	2.5	0.22	0.78825	0.25	0.25	0.28	0.98996
	0.5	0.06	5.0	0.663	0.5	0.531	0.25	5.0	0.15	0.69002	0.25	0.5	0.188	0.88983
**CAOV3**	0.1	0.63	2.5	0.824	0.5	0.69	0.1	2.5	0.19	0.56065	0.1	0.5	0.3	0.82677
	0.25	0.11	5.0	0.691	0.25	0.822	0.1	5.0	0.097	0.43884	0.25	0.5	0.05	0.77068
**OVCAR5**	0.01	0.66	1.0	0.807	0.25	0.864	0.05	1.0	0.36	0.64315	0.05	0.25	0.33	0.52942
	0.05	0.45	2.5	0.617	0.5	0.72	0.05	2.5	0.2	0.27683	0.05	0.5	0.27	0.45413

The cell lines were treated by two different concentrations of each drug. The CI was calculated using the Compusyn software(www.combosyn.com). The CI values indicate a synergistic effect when <1 based on the theorem of Chou and Talalay [21].

### LLL12B inhibited cell migration of ovarian cancer cells and enhanced the effect of cisplatin and paclitaxel

**Fig 4:**
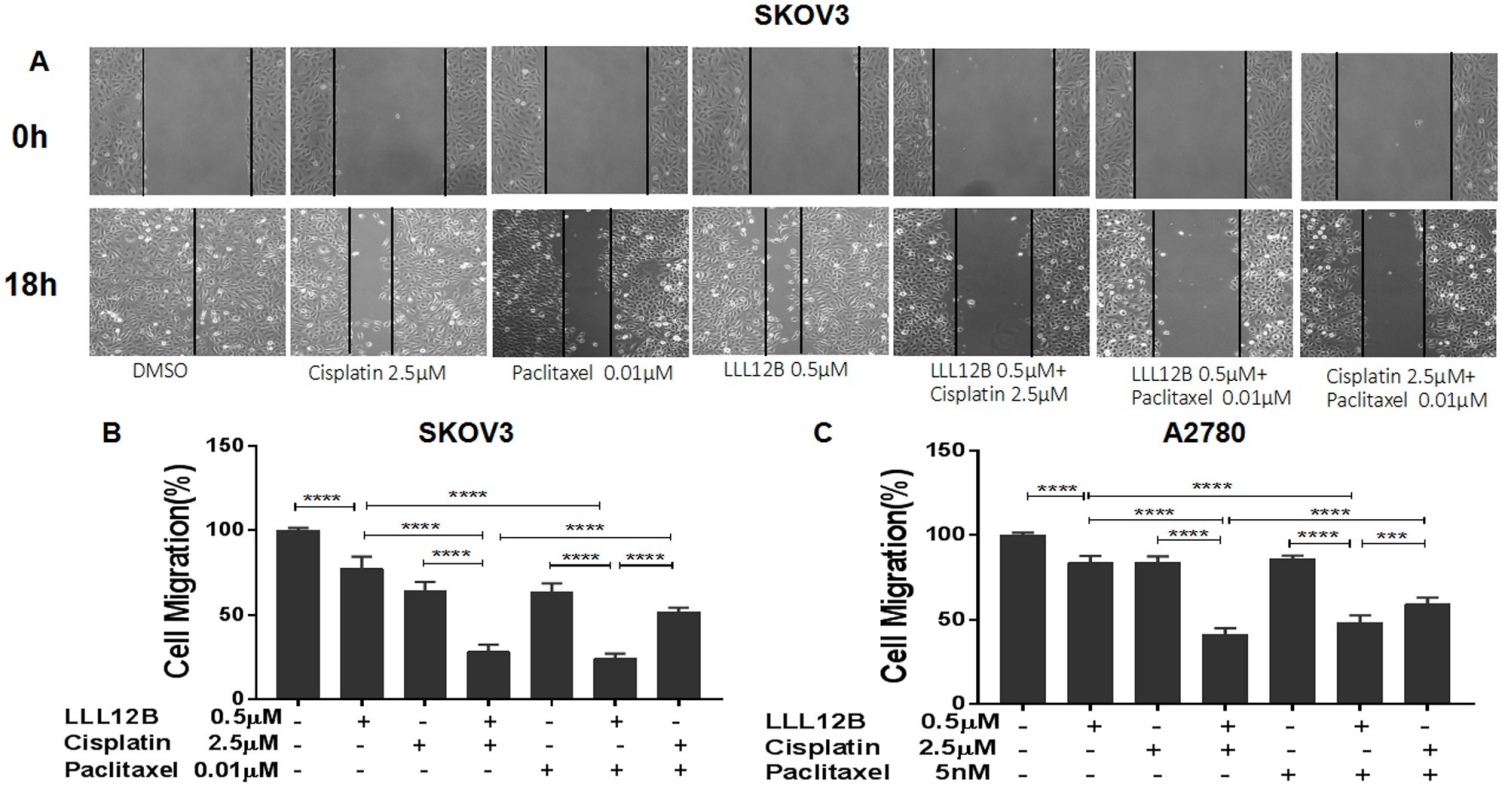
The effects of LLL12B, cisplatin, paclitaxel, and drug combination on cell migration. Wound-healing assays were performed to evaluate the migration ability of SKOV3 and A2780 ovarian cancer cells. SKOV3 and A2780 cells were seeded in 6-well plates and treated with DMSO or different concentration of drugs: LLL12B or cisplatin or paclitaxel or the combination. The differences were found to be significantly different at ***p<0.001 and ****p<0.0001.

Cell migration is an important step in tumor invasion and metastasis, which confers prognosis. According to previous literature reports [28], cancer cell migration can be inhibited by blocking of STAT3 pathway. Therefore, we tested the effects of the novel STAT3 inhibitor LLL12B on ovarian cancer cell migration and whether the effect of cisplatin and paclitaxel can be enhanced by LLL12B. Only A2780 and SKOV3 cell lines were tested because the monolayer phenotypes of the other two cell lines were not suitable for cell migration assays. Compared with the DMSO control, cell migration was inhibited by LLL12B. The combination of LLL12B with cisplatin or paclitaxel resulted in more significant inhibition of cell migration compared to monotherapy; notably, the inhibitory effects exceeded that of paclitaxel and cisplatin in combination (Fig 4).

These results indicate that LLL12B can inhibit cell migration of ovarian cancer cells, and may also enhance the effects of chemotherapy. This suggests that LLL12B may be helpful for treatment or prevention of ovarian cancer cell invasion and metastasis.

### LLL12B inhibited cell growth and enhanced the effect of cisplatin and paclitaxel

**Fig 5:**
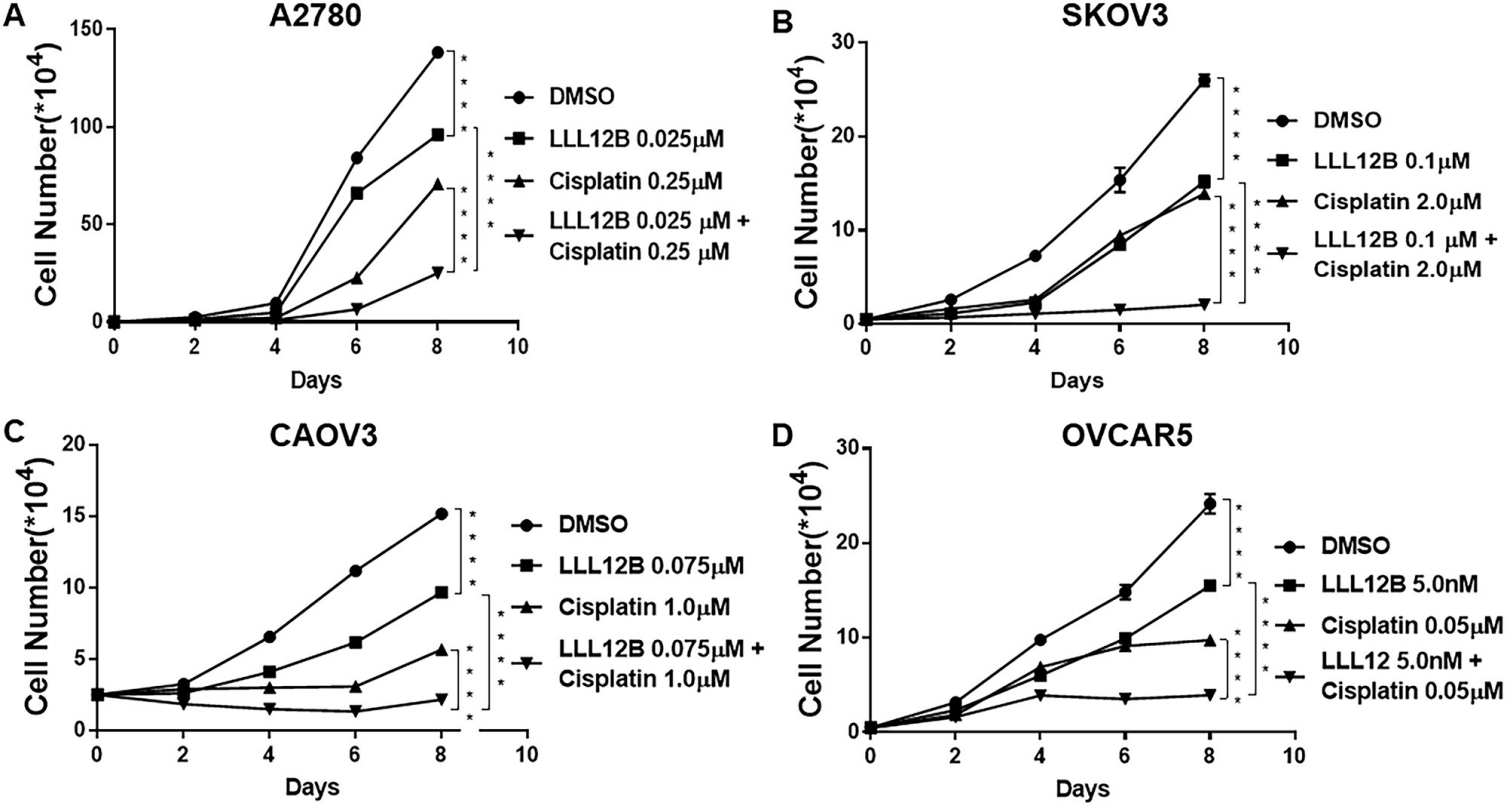
The effects of LLL12B, cisplatin, and drug combination on cancer cell growth. Cell growth assays were performed to evaluate cell proliferation ability of ovarian cancer cells. Cells were treated with LLL12B, cisplatin and their combination. The differences were found to be significantly different at **p<0.01 and ****p<0.0001. LLL12B alone or combined with cisplatin inhibited cell growth of ovarian cancer cells.

**Fig 6:**
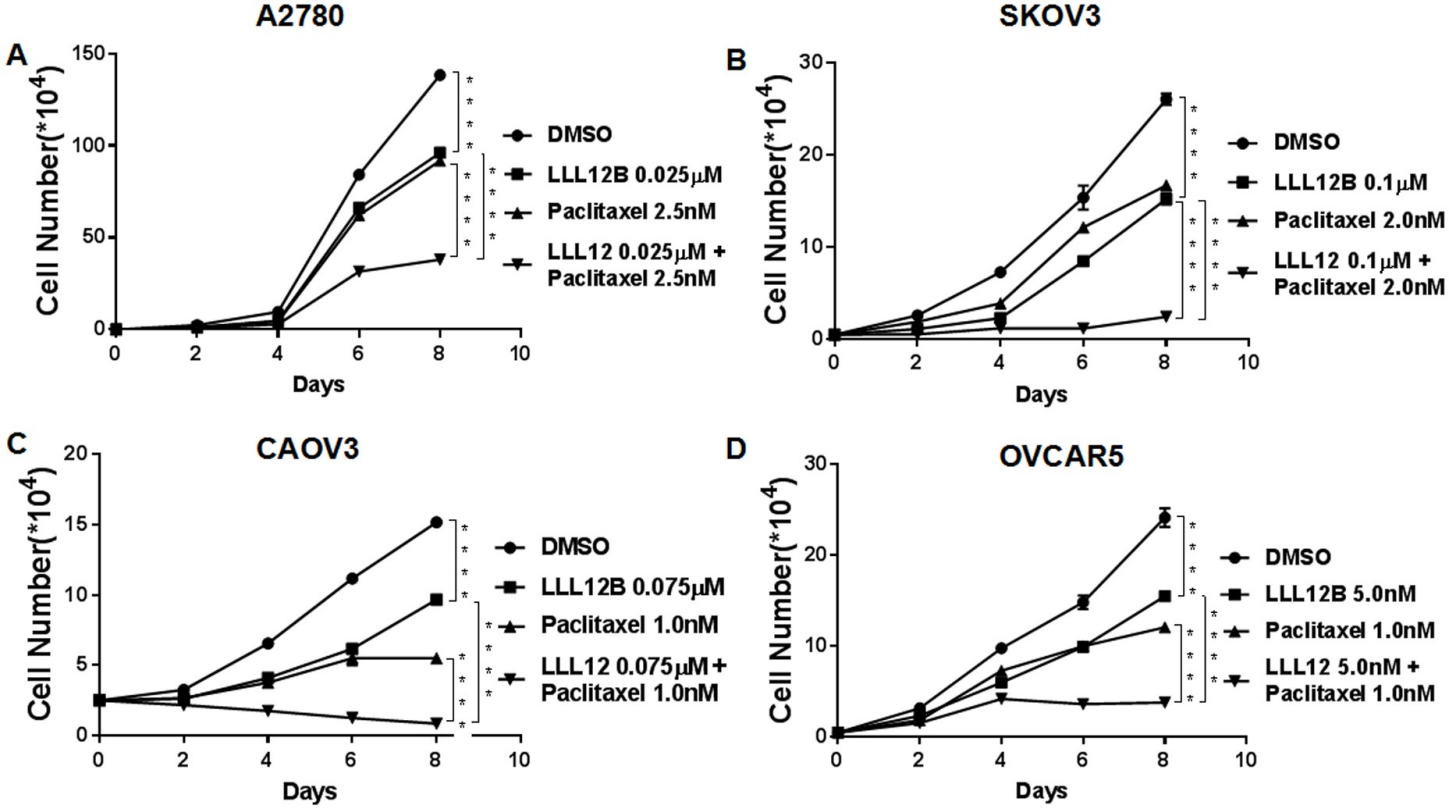
The effects of LLL12B, paclitaxel, and drug combination on cancer cell growth. Cell growth assays were performed to evaluate cell proliferation ability of ovarian cancer cells. Cells were treated with LLL12B, paclitaxel and their combination. The differences were found to be significantly different at **p<0.01 and ****p<0.0001. LLL12B alone or combined with paclitaxel inhibited cell growth of ovarian cancer cells.

Since LLL12B synergistically inhibited cell viability of ovarian cancer cells treated with cisplatin and paclitaxel, we then sought to investigate whether LLL12B could also inhibit cell growth using standard growth curves. Our results showed that LLL12B inhibited cell growth in all four ovarian cancer lines (Figs 5 and 6). Parallel to our observations for viability, combination treatment of LLL12B with cisplatin or paclitaxel resulted in greater inhibitory effects on cell growth than monotherapy (Figs 5 and 6).

## Discussion

Ovarian cancer is the tenth most common cancer and the fifth most lethal cancer of women in the United States [2, 3]. Among gynecologic malignancies, ovarian cancer has the highest mortality rate. Ovarian cancer is very difficult to diagnose early, and more than 50% of patients are diagnosed with advanced (stage III/IV) disease [2, 3]. The standard treatment for advanced ovarian cancer is radical resection combined with platinum-taxane combination chemotherapy [1, 29]. Despite small improvements in 5-year overall survival for this disease, there are still many challenges for its treatment. The 5-year overall survival of advanced-stage ovarian cancer is no more than 20–35%, and has not changed much for nearly twenty years. Both intrinsic and acquired chemoresistance remain problematic, and as many as 75% of ovarian cancer patients suffer from cancer recurrence [3]. It is therefore crucial to find new drugs to enhance the effect of current chemotherapy treatment. Targeted therapy often offers the benefit of precise action and fewer side effects.

As an important transducer of many cytokines and growth factors, STAT proteins have 7 members (STAT1, STAT2, STAT3, STAT4, STAT5A, STAT5B and STAT6), and STAT3 is the most widely known and researched [30]. Compared with normal tissues, STAT3 is overexpressed or constitutively activated in about 70% of human solid tumors and in 94% of ovarian cancers [31, 32]. A homodimer is formed when STAT3 is activated by phosphorylation., which translocates to the nucleus, recognizes and binds to STAT3-specific DNA-binding elements. Then some target genes can be regulated to promote cell growth, prevent apoptosis and so on [17, 33–35]. Abnormal activation of STAT3 can induce malignant cell transformation and is related to the poor prognosis of certain tumors. Conversely, the disruption of constitutively activated STAT3 can promote cell apoptosis and suppress tumor-cell growth. At the same time, it is reported that over expression of STAT3 also associated with cisplatin resistance and paclitaxel resistance [17, 36–38]. We have previously demonstrated that constitutive activation of STAT3 was present in ovarian cancer cell lines but not in normal ovarian surface epithelial cells [39], making selective STAT3 inhibition an excellent candidate for ovarian cancer treatment. In this study, we provide evidence that STAT3 inhibition may be a good enhancer for cisplatin and paclitaxel chemotherapy.

The SH2 domain is a critical module among the six structural domains of STAT3, which facilitate binding to specific p-Tyrosine (Tyr) motifs of receptors for activation of the protein [40, 41]. Interaction of the pTyr-SH2 domain with STAT3 dimerization represents an important molecular event for STAT3 functioning [40, 41]. For these reasons, most drugs have been designed to bind this domain. Many peptide-based inhibitors of STAT3 have been reported, but their use is limited by poor cell permeability and limited *in vivo* stability [42]. During recent years, many nonpeptide small molecule inhibitors have been developed, which show better stability [43, 44].

We previously developed several nonpeptide small molecule STAT3 inhibitors, such as LLL12, which inhibits STAT3 phosphorylation and suppresses the development of cancer [28, 45–47]. In the present study, we explored LLL12B, a carbamate-based prodrug for LLL12. LLL12B has one of the smallest molecular weights (374 dalton) compared to other STAT3 inhibitors. In addition, our *in vivo* pharmacokinetic studies in rats indicated that LLL12B is orally bioavailable (38.0%) and stable in the plasma, producing drug levels 5-fold better compared to LLL12 [20]. These results support that LLL12B is a superior drug relative to LLL12 to target STAT3 in ovarian cancer cells.

In this study, we tested LLL12B in several well-characterized human ovarian cancer cell lines. LLL12B consistently inhibited STAT3 phosphorylation and downregulated the downstream targets. STAT3 is one of the downstream target of Jak. In order to confirm that LLL12B is not a Jak inhibitor. We performed the WB assay at 2 hours and 4 hours after the addition of LLL12B. The results showed that (Fig 2C) p-STAT3 was inhibited in four cell lines, the p-Jak1 was only inhibited in A2780, the expression of Jak2 and Jak 1 in the other 3 cell lines were not detectable under the same protein concentration and treatment time. The results indicated that inhibition of p-STAT3 was the main function of LLL12B. That is, LLL12B is a STAT3 inhibitor nor a Jak inhibitor. Of couse, LLL12B might function a dual inhibitor of STAT3 and Jak1 in certain cell lines just like a few other STAT3 inhibitors [27].

LLL12B exerted potent inhibition of cell viability, migration and growth. When cisplatin or paclitaxel was combined with LLL12B, inhibition of these parameters was enhanced relative to monotherapy and, importantly, greater than that of paclitaxel with cisplatin, which currently represents the standard of care.

Paclitaxel is a microtubule-stabilizing drug and one of the main mechanisms that paclitaxel kills cancer cells is by a consequence of mitotic arrest. Cisplatin is a cytotoxic drug which kills cancer cells by damaging DNA and inhibiting DNA synthesis. The persistent activation of STAT3 signaling have been reported to confer resistance to paclitaxel and cisplatin in cancer cells including ovarian cancer cells [33–35]. STAT3 may confer the resistance to paclitaxel and cisplatin by inducing its downstream targets such as Survivin, C-Myc, Cyclin D1, and others.

Therefore, WB assay was performed by us, compared to the combination of LLL12B with cisplatin or/and paclitaxel or LLL12B alone, cisplatin or paclitaxel alone are in general not able to significantly inhibit Survivin, C-Myc, Cyclin D1 as shown in Fig 2B. Inhibiting downstream targets such as Survivin, C-Myc, Cyclin D1, and others by LLL12B, is likely one of the main mechanisms of sensitizing ovarian cancer cells to cisplatin or paclitaxel and likely one the main mechanisms of synergism by drug combination. Additional study in the future will be needed to further elucidate the more detailed mechanism of action of synergism by LLL12B + cisplatin or LLL12B + paclitaxel in ovarian cancer cells.

In conclusion, the novel small molecule STAT3 inhibitor, LLL12B, designed by AMLSD methodology shows excellent therapeutic potential in ovarian cancer cell lines. Our results suggest that LLL12B is a potent STAT3 inhibitor in ovarian cancer, and that LLL12B in combination with the current front-line chemotherapeutic drugs cisplatin and paclitaxel may represent a promising approach for ovarian cancer therapy that warrants further study.

## Supporting information

S1 Raw images (PDF)
