# Supplementary material for: A novel small molecule LLL12B inhibits STAT3 signaling and sensitizes ovarian cancer cell to paclitaxel and cisplatin
Source: PLoS One. 2021 Apr 28;16(4):e0240145. doi: 10.1371/journal.pone.0240145 (PMC8081214; doi:10.1371/journal.pone.0240145)

LLL12B(μM) 0 0.25 0.5 1.0

A2780

p-STAT3(86)

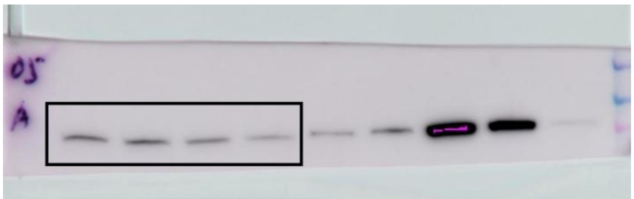

Amersham Imager 680

STAT3(86)

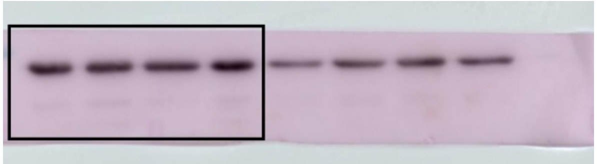

Amersham Imager 680

C-myc(51)

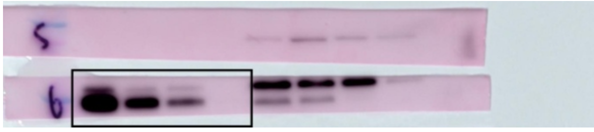

Amersham Imager 680

CyclinD1(36)

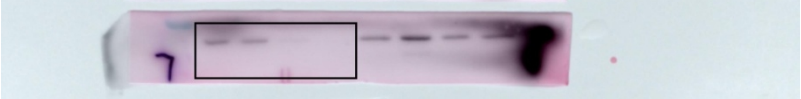

Amersham Imager 680

Survivin(16)

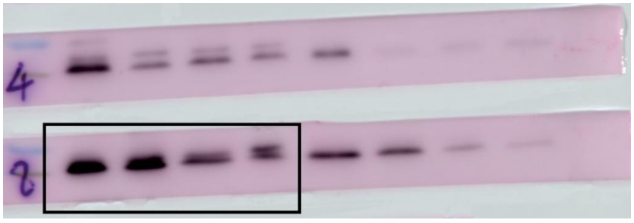

Amersham Imager 680

GAPDH(36)

Amersham Imager 680

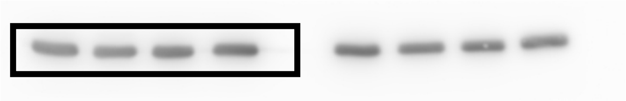

Cleaved caspase3(19) LLL12B(μM) 1.0 0.5 0.25 **0**

Storm scanner

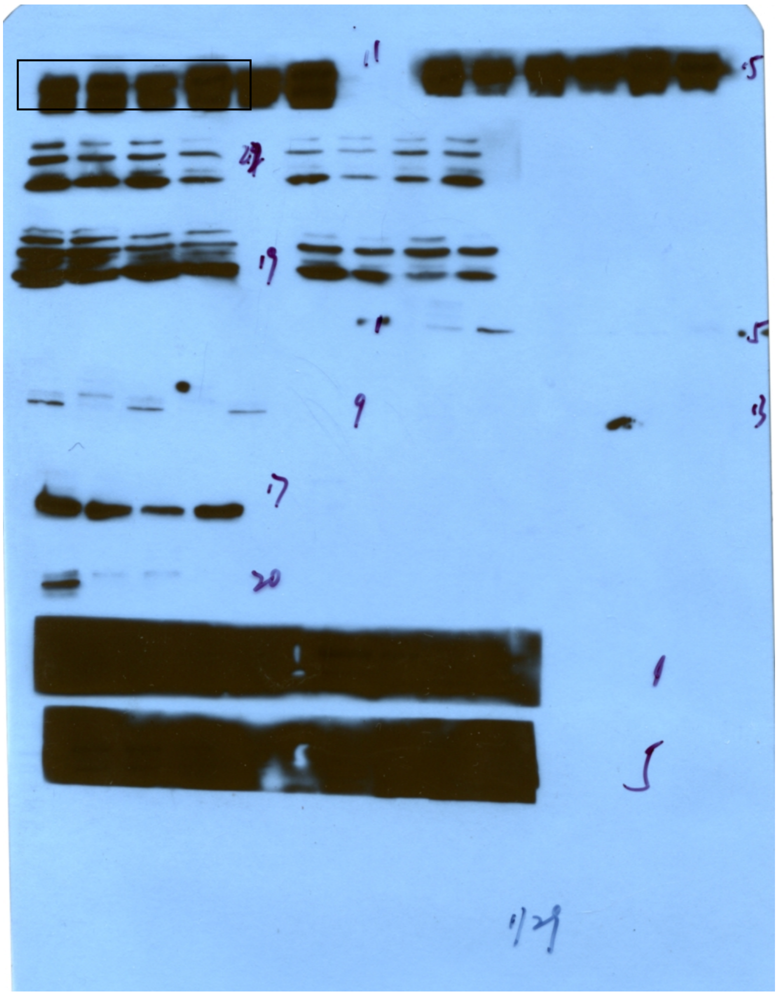

skov3

LLL12B(μM) 0 0.5 1.0 2.5

p-STAT3(86)

Amersham Imager 680

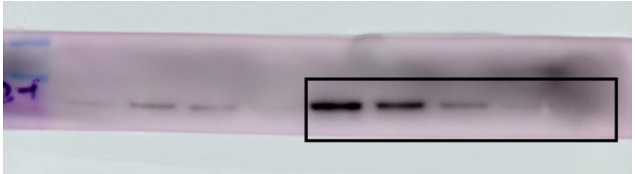

STAT3(86)

Amersham Imager 680

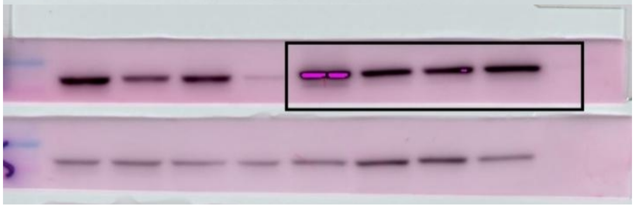

CyclinD1(36)

Amersham Imager 680

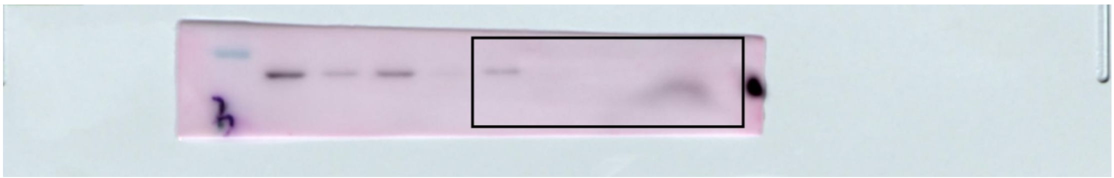

Survivin(16)

Amersham Imager 680

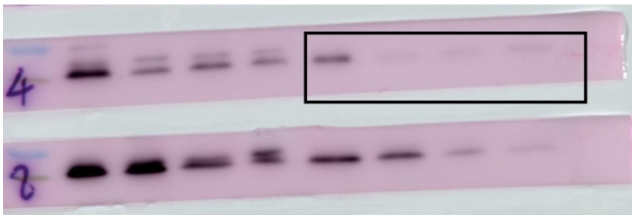

Cleaved caspase3(19)

Amersham Imager 680

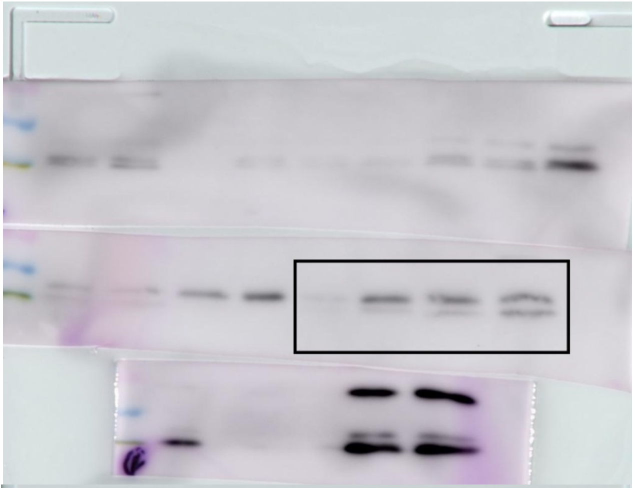

GAPDH(36)

Amersham Imager 680

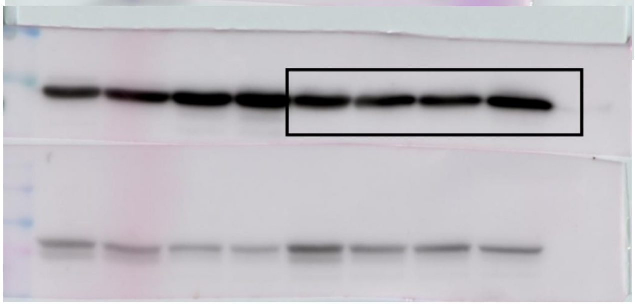

C-myc(51)

Storm scanner

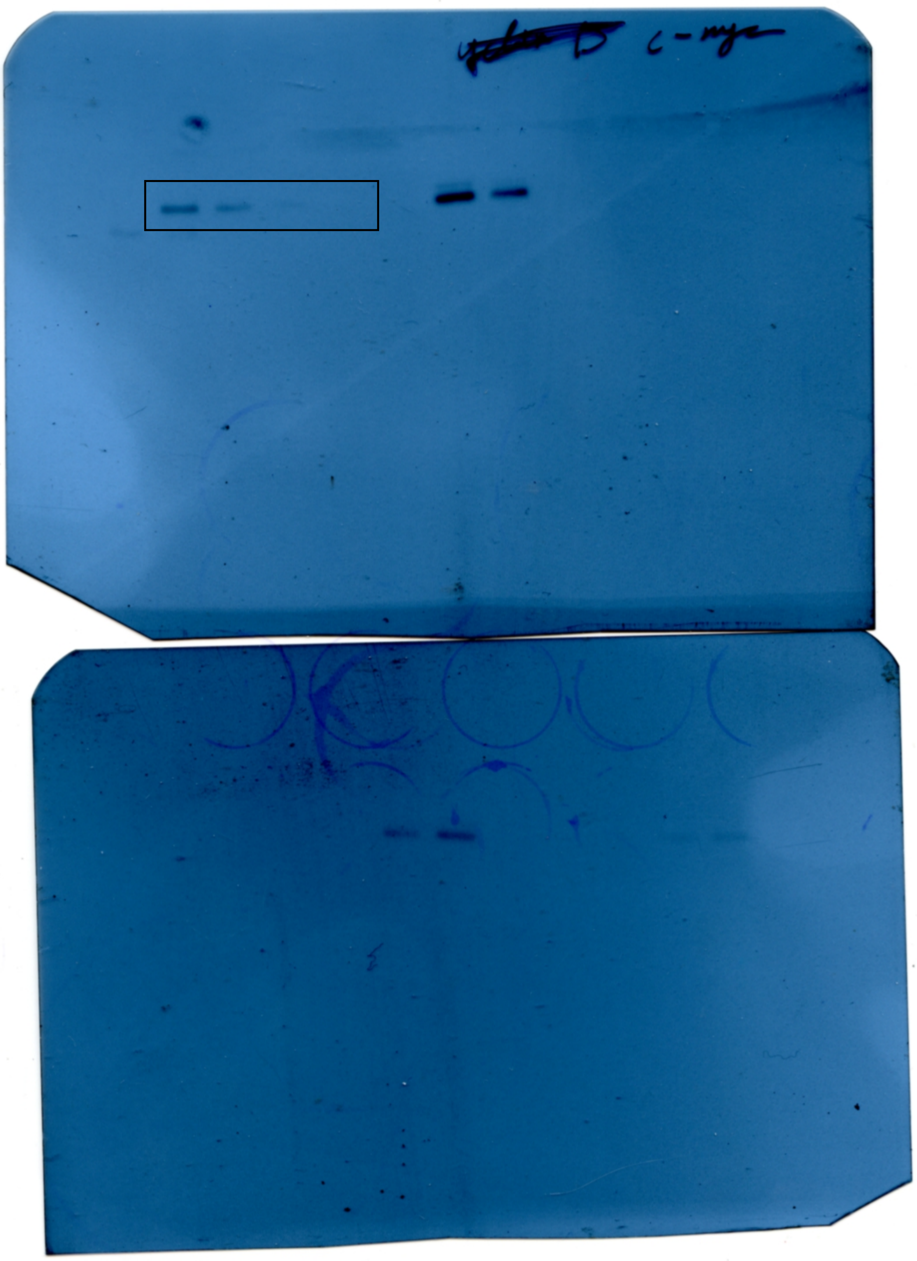

LLL12B(μM) 0 0.5 1.0 2.5

CAOV3

p-STAT3(86)
Amersham Imager 680

STAT3(86)
Amersham Imager 680

c-myc(51)
Amersham Imager 680

Survivin(16)
Amersham Imager 680

cleaved caspase3(19)
Amersham Imager 680

GAPDH(36)
Amersham Imager 680

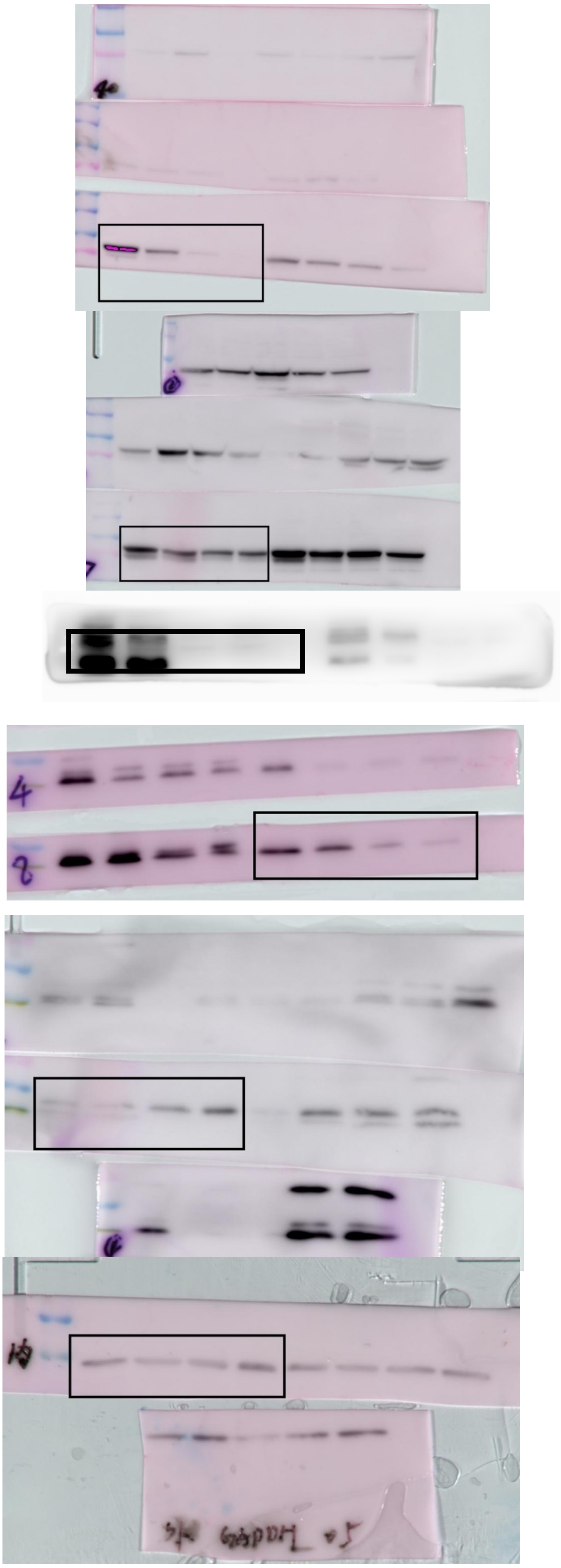

cyclinD1(36)

Storm scanner

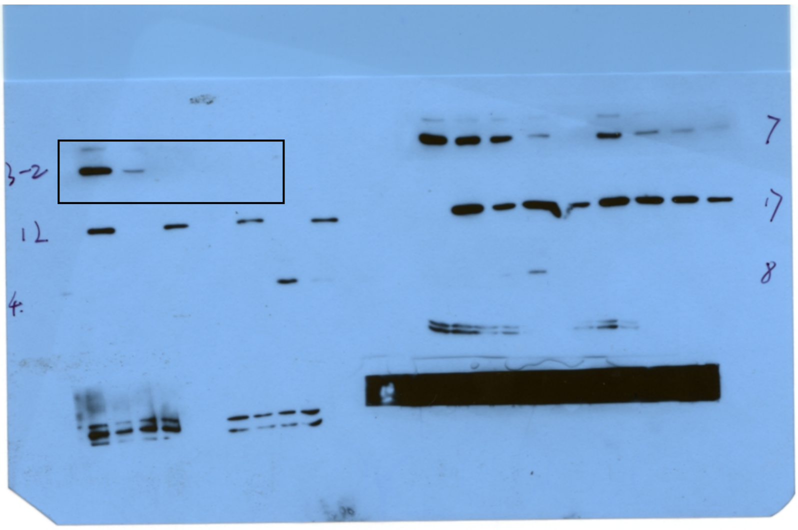

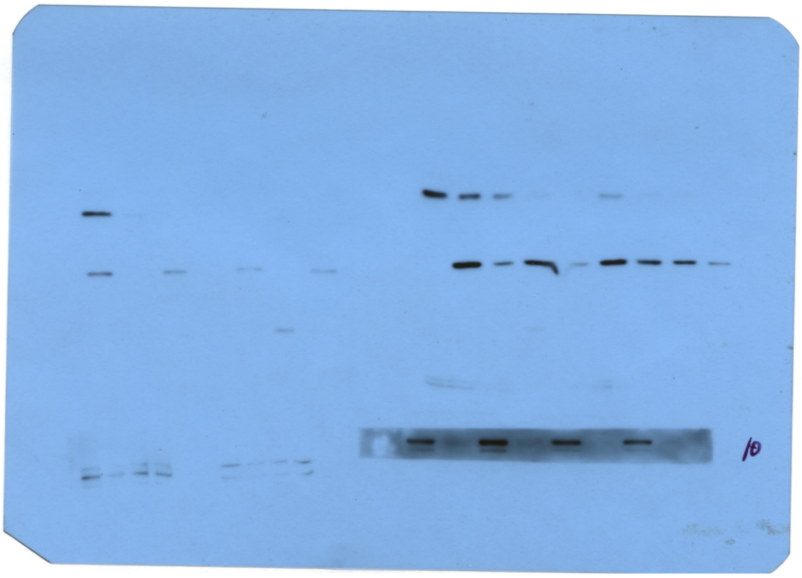

LLL12B(μM) 0 0.1 0.25 0.5

OVCAR5

p-STAT3(86)
Amersham Imager 680

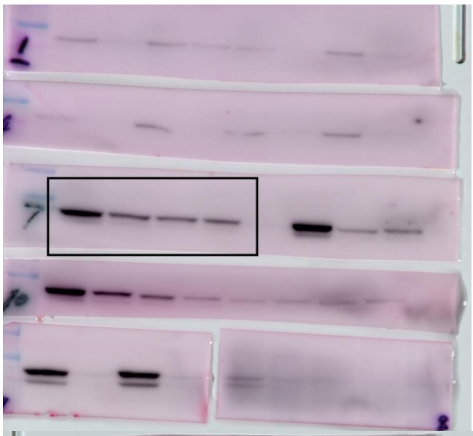

STAT3(86)
Amersham Imager 680

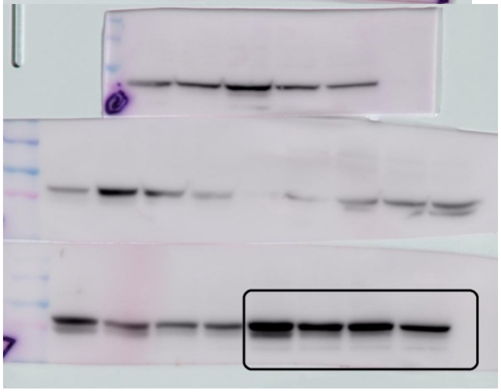

survivin(16)
Amersham Imager 680

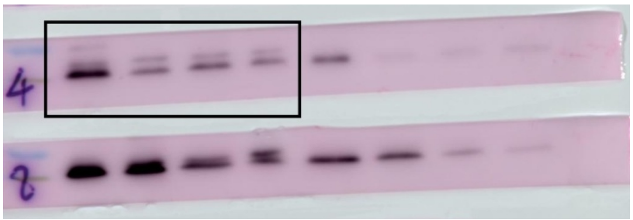

cleave caspase3(19)
Amersham Imager 680

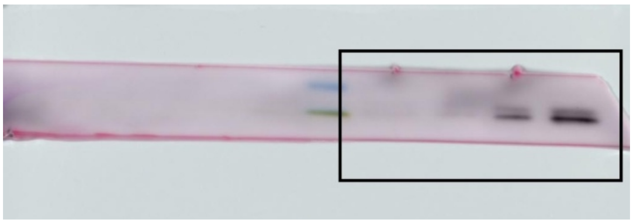

GAPDH(36)
Amersham Imager 680

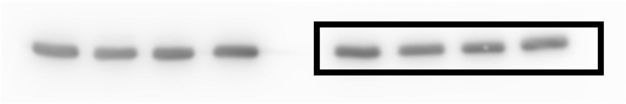

C-myc(51)

Storm scanner

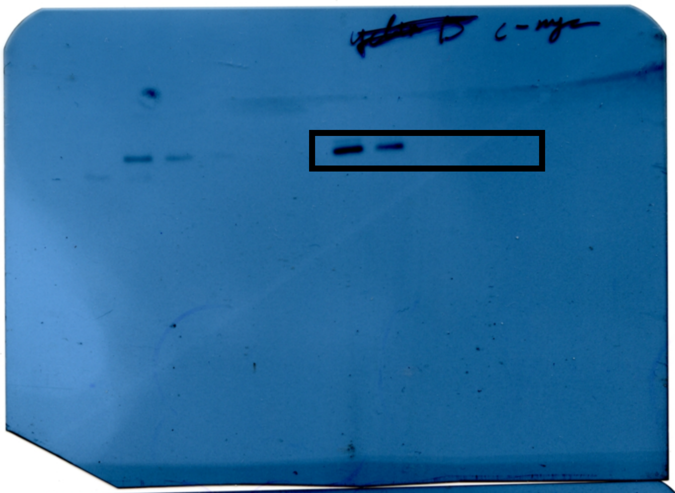

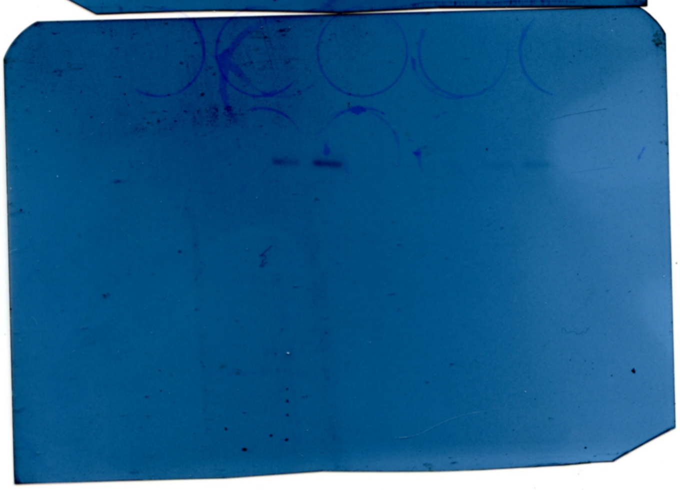

cyclinD1(36)

Storm scanner

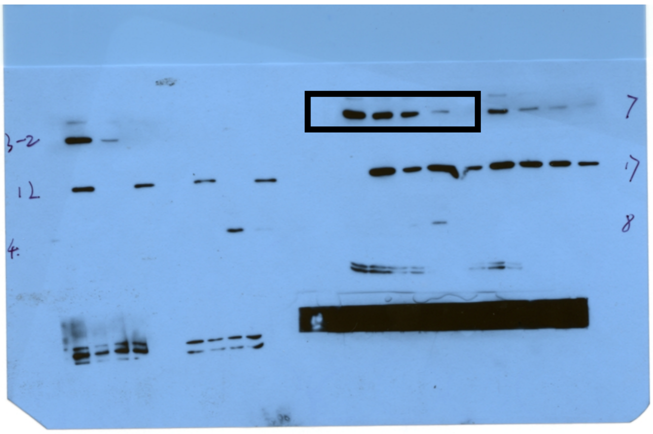

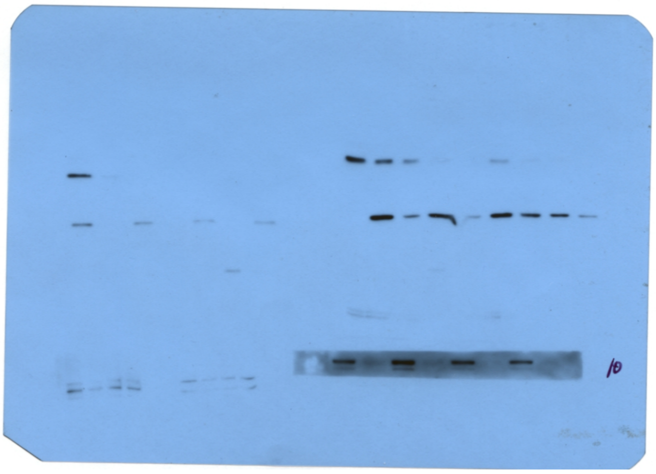

DMSO L0.5 C2.5 L+C P0.5 L+P

CAOV3

p-STAT3(86)
Amersham Imager 680

c-myc(51)
Amersham Imager 680

survivin(16)
Amersham Imager 680

cleaved caspase3(19)
storm scanner

GAPDH(36)
Amersham Imager 680

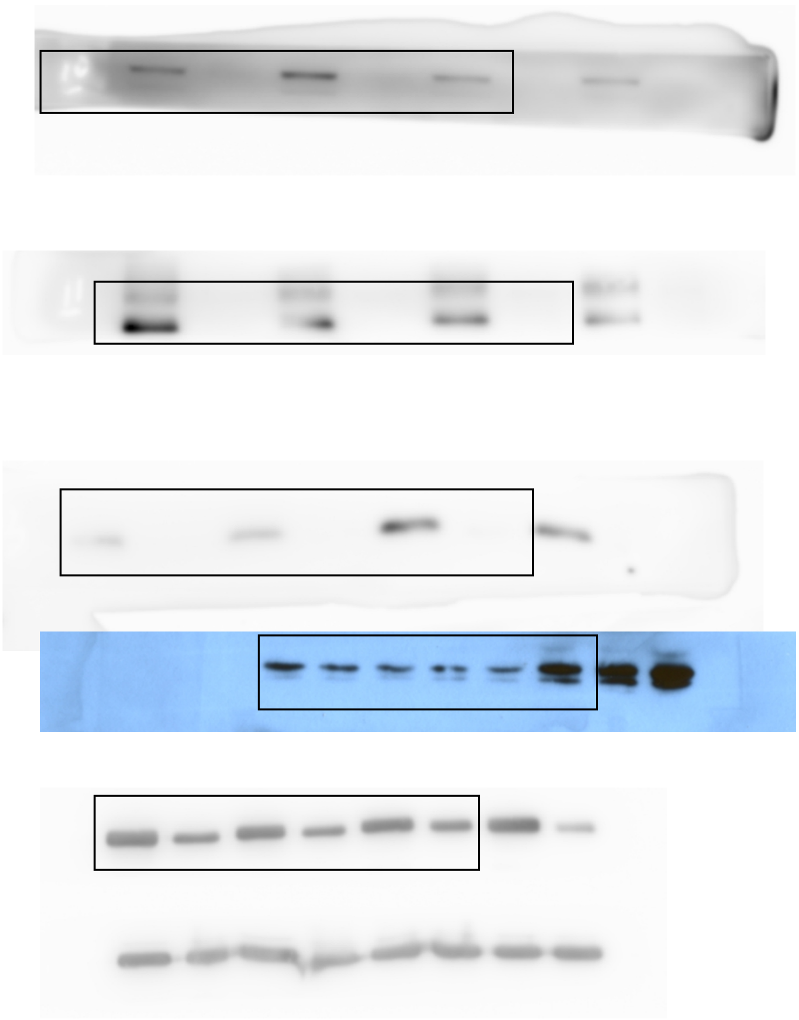

STAT3(86)

Storm scanner

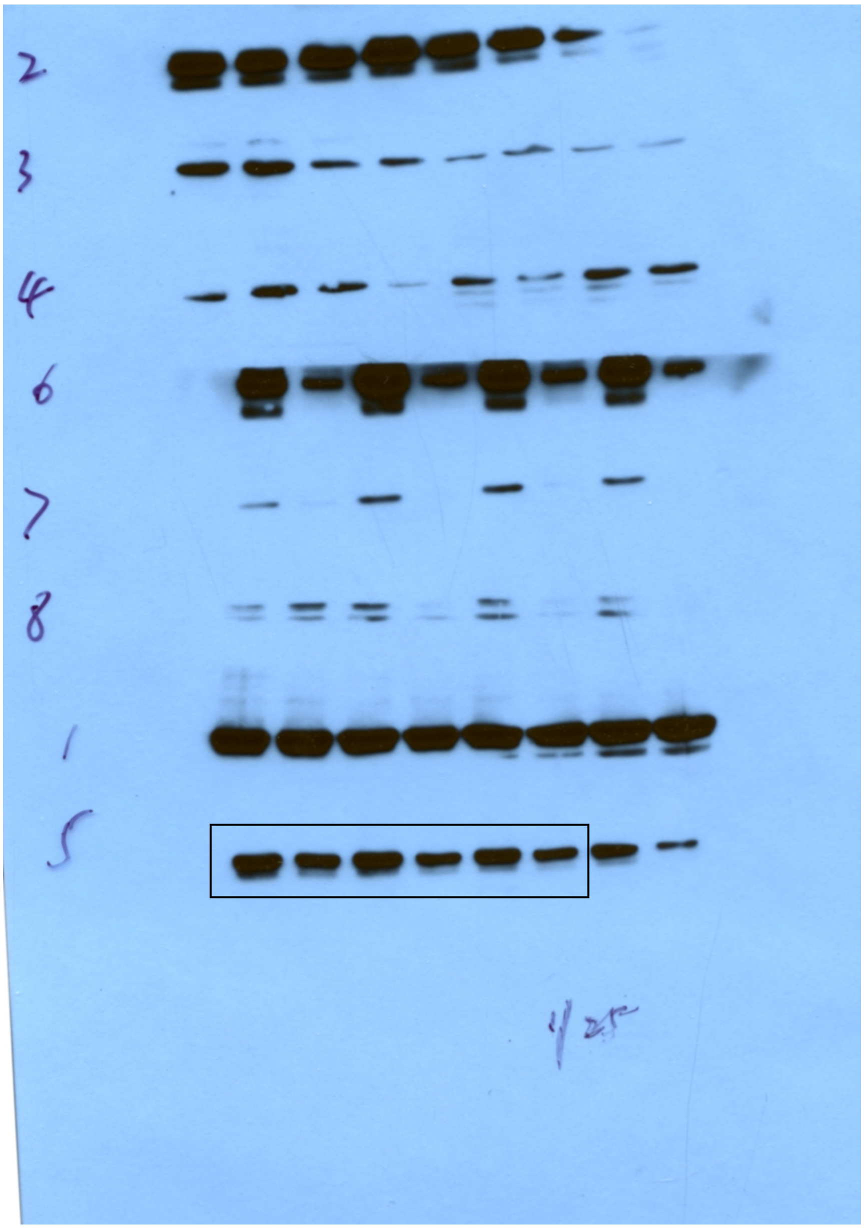

cyclinD1(36)

Storm scanner

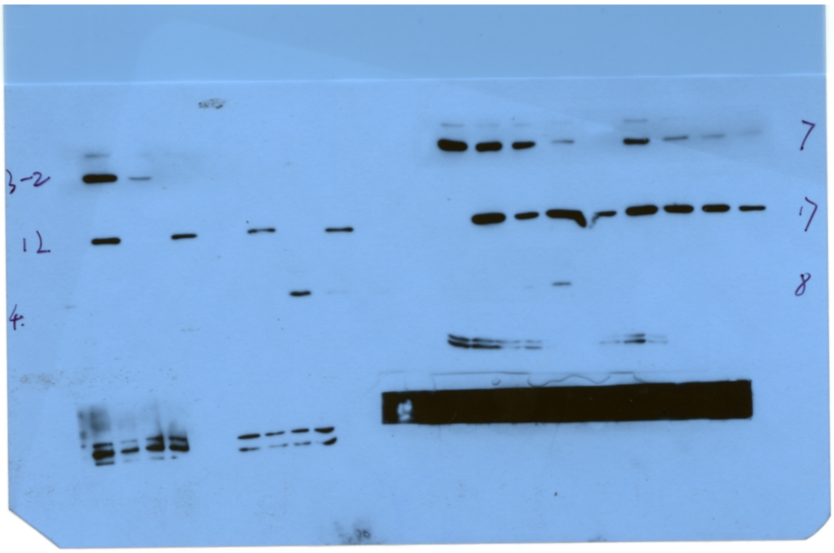

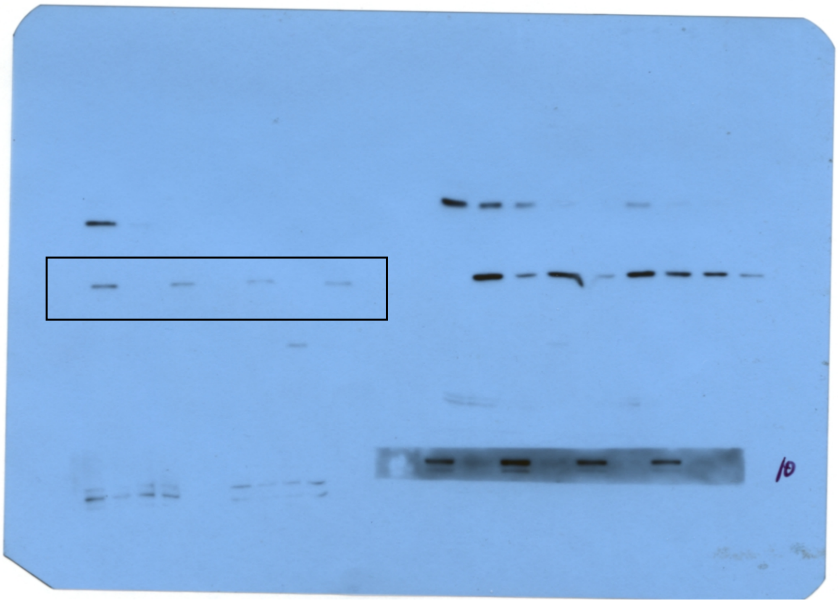

DMSO L0.1 C 2.5 L+C P0.25 L+P

OVCAR5

p-STAT3(86)

Amersham Imager 680

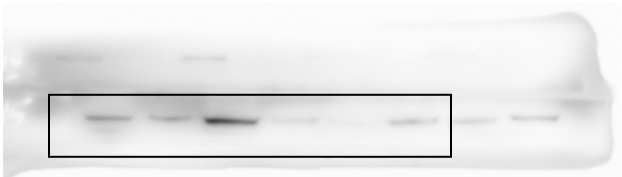

c-myc(51)

Amersham Imager 680

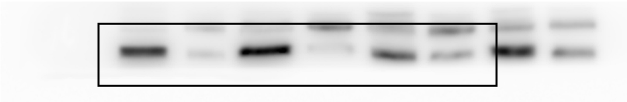

survivin(16)

Amersham Imager

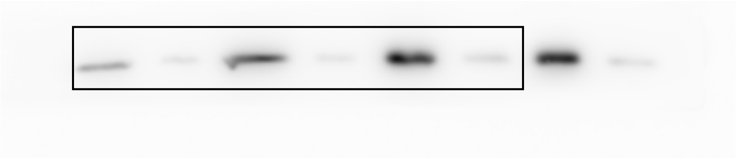

680

cleaved caspase3(19)

Storm scanner

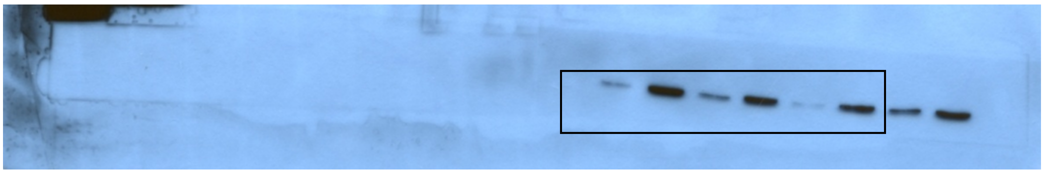

GAPDH(36)

Amersham Imager 680

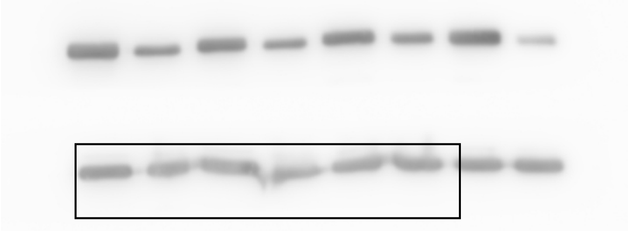

STAT3(86)

Storm scanner

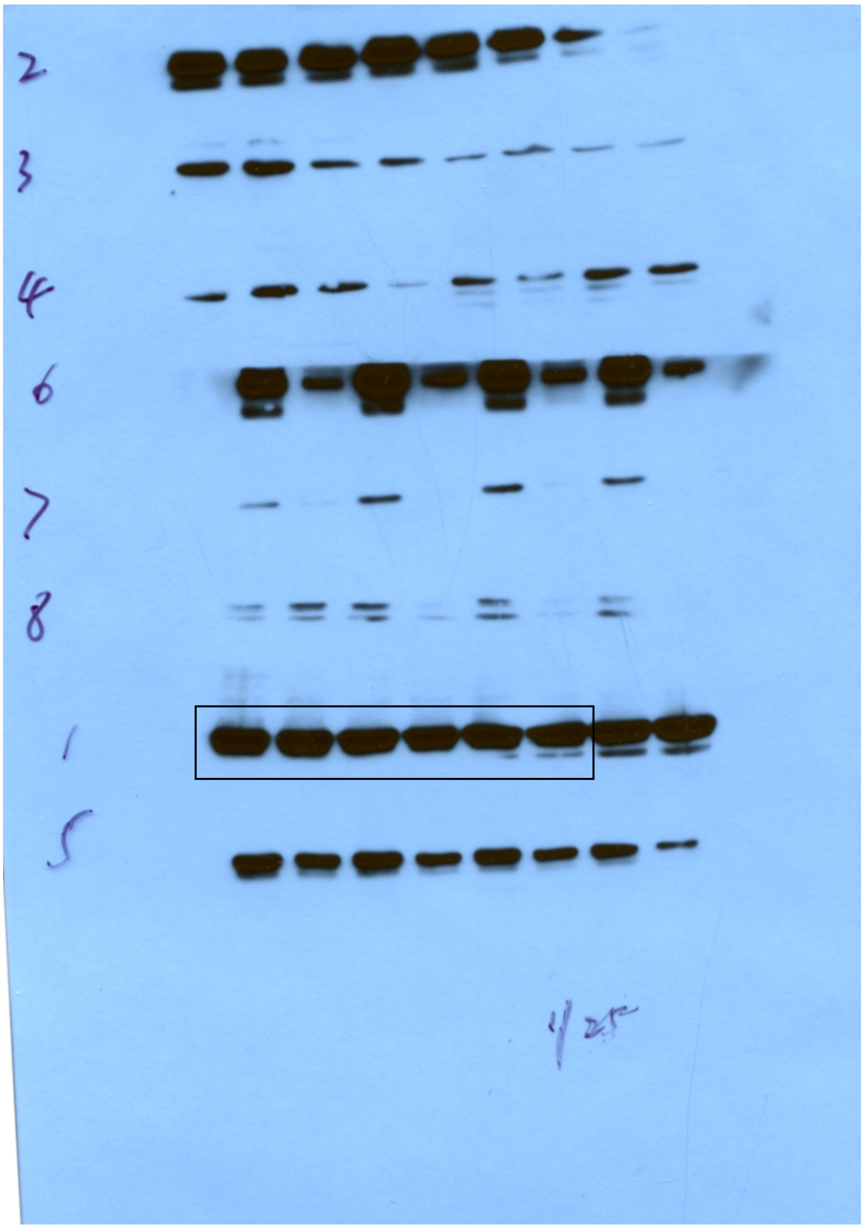

cyclinD1(36)

Storm scanne

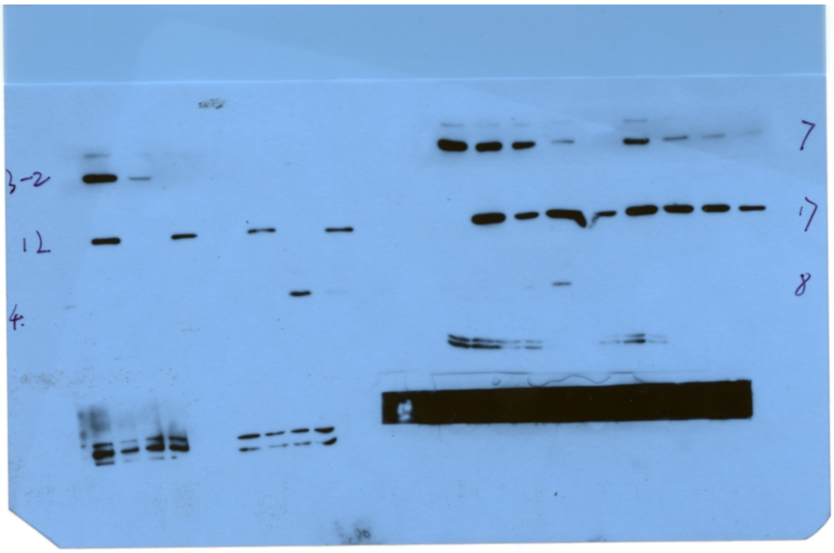

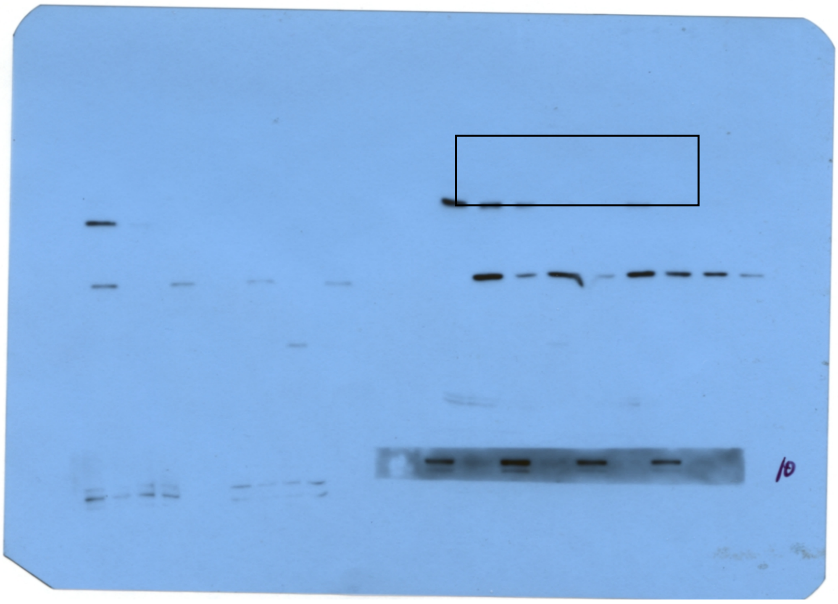

DMSO L0.5 C2.5 L+C P0.01 L+P

SKOV3

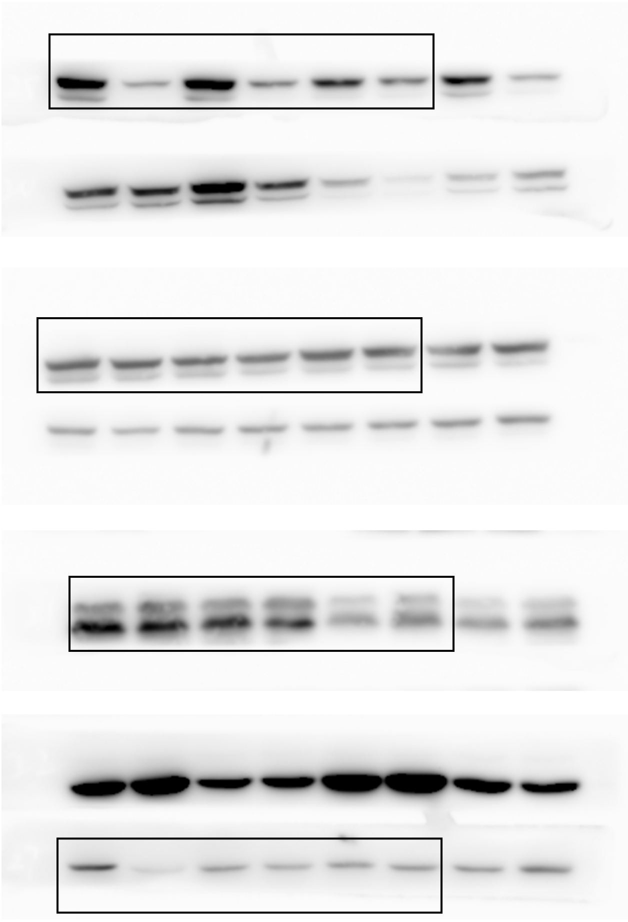

p-STAT3(86)

Amersham Imager 680

STAT3(86)

Amersham Imager 680

c-myc(51)

Amersham Imager 680

cyclinD1(36)

Amersham Imager 680

Storm scanner

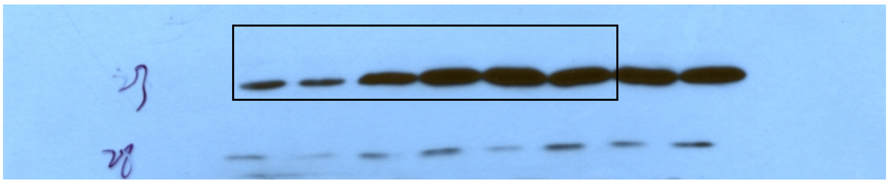

GAPDH(36)

Survivin(16)

Storm scanner

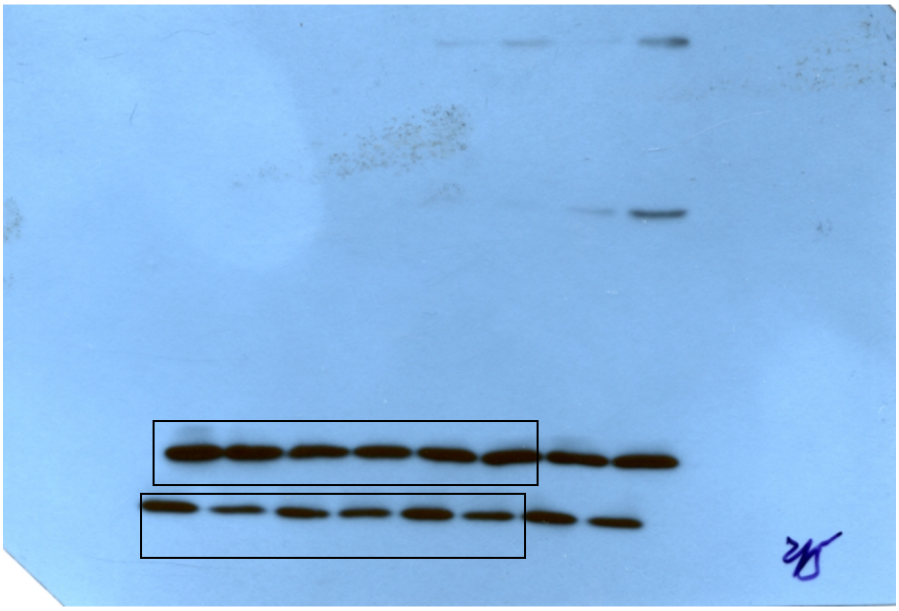

Supplement: S1 Raw images — (PDF) [file pone.0240145.s001.pdf]
